# Polymorphism of E6 and E7 Genes in Human Papillomavirus Types 31 and 33 in Northeast China

**DOI:** 10.1155/2023/9338294

**Published:** 2023-03-13

**Authors:** Miao Yu, Si Wu, Shuang Wang, Changwan Cui, Yiping Lu, Zhengrong Sun

**Affiliations:** Department of BioBank, Shengjing Hospital of China Medical University, Shenyang, Liaoning, China

## Abstract

Persistent infection with human papillomavirus (HPV) types 31 and 33 is an important causative factor for cervical cancer. The E6/E7 genes are key oncogenes involved in the immortalization and transformation of human epithelial cells. Genetic polymorphism may lead to differences in the virus' carcinogenic potential, the immune reaction of the host, and the potencies of vaccines. Few studies on HPV31/33 E6/E7 genetic polymorphism have been carried out. To study the genetic polymorphism of HPV31 and HPV33 E6/E7 genes in northeast China, these genes (HPV31 E6/E7, *n* = 151; HPV33 E6/E7, *n* = 136) were sequenced and compared to reference sequences (J04353.1, M12732.1) using BioEdit. Phylogenetic trees were constructed by the neighbor-joining method using MegaX. The diversity of the secondary structure was estimated using the PSIPred server. The positively selected sites were analyzed using PAML4.9. The major histocompatibility complex (MHC) class I and MHCII epitopes were predicted using the ProPred-I server and ProPredserver. B-cell epitopes were predicted using the ABCpred server. In the 151 HPV31E6 sequences, 25 (25/450) single-nucleotide mutations were found, 14 of which were synonymous mutations and 11 were nonsynonymous. In the 151 HPV31E7 sequences, 8 (8/297) nucleotide mutations were found, 3 of which were synonymous mutations and 5 were nonsynonymous. In the 136 HPV33E6 sequences, 17 (17/450) nucleotide mutations were observed, 7 of which were synonymous mutations and 10 were nonsynonymous. C14T/G (T5I/S) was a triallelic mutation. Finally, in the 136 HPV33E7 sequences, 9 (9/294) nucleotide mutations were observed, 3 of which were synonymous mutations and 6 were nonsynonymous. C134T/A (A45V/E) and C278G/A (T93S/N) were triallelic mutations. Lineage A was the most common lineage in both HPV31 and HPV33. In all of the sequences, we only identified one positively selected site, HPV33 E6 (K93N). Most nonsynonymous mutations were localized at sites belonging to MHC and/or B-cell predicted epitopes. Data obtained in this study should contribute to the development and application of detection probes, targeted drugs, and vaccines.

## 1. Introduction

Cervical cancer is the most common genital malignancy among females. Nearly 530,000 cases of it are newly diagnosed every year globally [1], more than 130,000 of which are in China. It is widely accepted that persistent infection with high-risk human papillomavirus (HR-HPV) is the most important risk factor for cervical cancer [2]. The risk of developing cervical cancer in HPV-infected patients is 50-fold higher than that in uninfected women [3, 4]. High-risk human papillomavirus (HR-HPV) includes 15 different types: HPV16, HPV18, HPV31, HPV33, HPV35, HPV39, HPV45, HPV51, HPV52, HPV56, HPV58, HPV59, HPV68, HPV73, and HPV82. Each type can also be divided into multiple sublineages according to genetic polymorphism. Studies have shown that the distribution of different sublineages of the same type of HR-HPV differs significantly among age groups and regions, which may lead to the corresponding discrepancies in cervical lesions in the infected population [5-7]. Furthermore, genetic variations of HR-HPV show ethnic and geographical differences [8, 9]. Some of these genetic variations can change the amino acid sequences, which can in turn affect the carcinogenic potential of the virus, the immune reaction of the host, and the potency of vaccines. The proteins E6 and E7, which are expressed at an early stage of viral infection, are the oncoproteins that have been studied most in HR-HPV. They are the key oncoproteins involved in the immortalization and transformation of human epithelial cells and act through their interplay with multiple host proteins [10].

The *p*53 tumor suppressor protein is a main target of E6. E6 also activates telomerase expression and regulates the activity of PDZ domain-containing and tumor necrosis factor receptors [11]. E7 proteins have been deemed classic targets of the retinoblastoma (Rb) family of proteins. These proteins control the activity of E2F transcription factors' degradation, leading to an increase in the expression of E2F-responsive genes. Some researchers have shown that knocking down the expression of E6 and E7 in cervical cancer cells can suppress their growth and induce apoptosis [12]. Therefore, HR-HPV E6 and E7 are potential targets for diagnosis and therapeutic vaccine design [13].

In summary, the genetic polymorphism of the E6 and E7 genes in HR-HPV warrants considerable attention. A large number of studies have also shown that HPV16 and HPV18 E6 and E7 gene polymorphisms are of great significance for the biological function of viruses and related disease progression. HPV31 and HPV33 are particularly prevalent in Western countries rather than Asian ones. They also have strong carcinogenic potential. Several nonsynonymous mutations have been found in HPV31/HPV33E6 and E7 globally as well as in southwest China. These nonsynonymous mutations may affect the structures and functions of proteins by changing their amino acid sequences, thereby affecting the viability of viruses, their carcinogenic potential, and their interactions with host cells. This may in turn affect the detection of viruses, the treatment of virus-related diseases, and the effectiveness of vaccines. Research has also shown that sublineages of HPV31 and HPV33 show differences in E6 and E7 depending on the geographical distribution. However, there have been few studies on the genetic polymorphism of the E6 and E7 genes in HPV31 and HPV33 found in northeast China. Therefore, in this study, we used PCR amplification to identify single-nucleotide polymorphisms of the E6 and E7 genes in HPV31 and HPV33 in northeast China. Then, we analyzed their genetic polymorphisms, intratypic variations, phylogeny, and positive selection to characterize the distribution of polymorphism in the E6 and E7 genes in HPV31 and HPV33 in northeast China. Although the available data are still limited, our results provide robust information that will contribute to the development of diagnostic probes, targeted therapies, and vaccines based on the E6 and E7 genes in HPV31 and HPV33.

## 2. Materials and Methods

### 2.1. Ethics Statement

This study was approved by the Ethics Committee of Shengjing Hospital of China Medical University (Shenyang, China). All participants provided informed consent before samples were collected. The privacy of all subjects was carefully protected.

### 2.2. Clinical Sample Collection

Between March 2018 and February 2019, we collected cervical samples from women who underwent cervical screening at Shengjing Hospital of China Medical University. Then, the HPV-DNA was extracted and examined by the virus laboratory of Shengjing Hospital of China Medical University. After that, we collected HPV31-positive DNA samples and HPV33-positive DNA samples for further study. In the present study, 204 samples that were HPV31-positive (median age 41 years; range 18–69) and 199 samples that were HPV33-positive (median age 43 years; range 19–80) were collected.

### 2.3. PCR Amplification and Sequencing

The E6 and E7 genes of HPV31 and HPV33 were amplified by nested PCR with two pairs of specific primers designed using Primer 5.0 bioinformatic software, in accordance with the published GenBank reference sequences (accession no. J04353.1 https://www.ncbi.nlm.nih.gov/nuccore/J04353.1 and accession no. M12732.1 https://www.ncbi.nlm.nih.gov/nuccore/M12732.1, respectively). The specific primers of HPV31 and HPV33 are listed in Table 1. The PCR amplification of each gene was first performed in a 25 *µ*l reaction volume containing 3.0 *μ*l of extracted DNA, 12.5 *μ*l of GoTaq® Green Master Mix, 2×, 1.0 *μ*l each of primers F1 and R1, and 7.5 *μ*l of nuclease-free water. PCR reactions were preheated for 2 min at 95°C; followed by 30 cycles of 95°C for 30 s, 55°C for 30 s, and 72°C for 90 s; and then a final extension step at 72°C for 10 min. Next, we used the products of the previous step to perform a second amplification in a 50 *µ*l reaction volume containing 2.0 *μ*l of products of the previous step, 25 *μ*l of GoTaq® Green Master Mix, 2×, 2.0 *μ*l each of primers F2 and R2, and 19 *μ*l of nuclease-free water. These reactions were performed by the same procedure as described above. Finally, the PCR products were visualized by 1% agarose gel electrophoresis and sent to Invitrogen for sequencing.

### 2.4. Variant Analysis and Phylogenetic Tree Construction

All nucleotide sequences containing E6 and E7 were aligned and compared with the corresponding reference sequences HPV31J04353.1 and HPV33 M12732.1, respectively, by BioEdit. The nucleotide sequences were translated into proteins using MEGAX Alignment Explorer for the determination of the amino acid changes caused by nucleotide polymorphism. The secondary structures of the reference proteins were predicted using the PSIPred server (https://bioinf.cs.ucl.ac.uk/psipred/) with the default settings. Neighbor-joining phylogenetic trees of the E6 and E7 variants in HPV31 and HPV33 were subsequently constructed using MEGAX with the Maximum Composite Likelihood model. The tree topology was evaluated by employing bootstrap resampling 1000 times. The reference sequences used in this study to construct the phylogenetic branches were obtained from the GenBank sequence database, as described in previous studies. Numbers above the branches indicate the bootstrap values. All data were confirmed by repeating the PCR amplification and sequence analyses at least twice.

### 2.5. Selective Pressure Analysis

To identify sites potentially undergoing positive selection in the E6 and E7 genes of HPV31 and HPV33, the CodeML program in PAML 4.9 software (Phylogenetic Analyses by Maximum Likelihood, https://abacus.gene.ucl.ac.uk/software/paml.html) was used. This program performed likelihood ratio tests (LRTs) to infer nonsynonymous and synonymous nucleotide divergence for coding regions, employing the method described by Nei and Gojobor [14, 15]. The proteins encoded by the E6 and E7 genes of HPV31 and HPV33 were aligned by MegaX.

### 2.6. Epitope Prediction

The human leukocyte antigen (HLA) class I binding promiscuous epitopes were predicted using the ProPred-I server (https://www.imtech.res.in/raghava/propred1/) [16] with the default settings. Epitopes for HLA class II alleles were predicted using the ProPred server (https://www.imtech.res.in/raghava/propred/) [17] with the default settings. B-cell epitopes in the E6 and E7 genes were predicted using the ABCpred server (https://www.imtech.res.in/raghava/abcpred/ABC_submission.html), with the default settings [18].

### 2.7. Statistical Analysis

The distributions of the identified variants associated with cervical disease were assessed by Fisher's exact test. *P* < 0.05 was considered to represent a statistically significant difference.

## 3. Results

Among all of the HPV31 and HPV33 samples, 151 sequences of the E6 and E7 genes of HPV31 and 136 sequences of those of HPV33 were amplified and sequenced successfully. The failed cases may have occurred because of the small number of copies of infected HPV in host cells and the limited amplicons obtained for sequencing.

### 3.1. Genetic Polymorphism of HPV31 E6 and E7

Upon comparison with the HPV31 reference sequence (J04353.1), 16 (10.60%) isolates were completely identical in the E6 gene, while the remaining 135 (89.40%) showed single-nucleotide variations. We found 25 (25/450) nucleotide positions that varied from the reference sequence. 14 (56.00%) of these were synonymous substitutions, while 11 (44.00%) were nonsynonymous, leading to amino acid changes. The isolates were divided into 20 subtypes named HPV31E601–HPV31E620 based on their mutations. HPV31E601 showed complete homology with the reference. HPV31E609–HPV31E614, HPV31E616–HPV31E618, and HPV31E620 were discovered for the first time.

**Figure 1:**
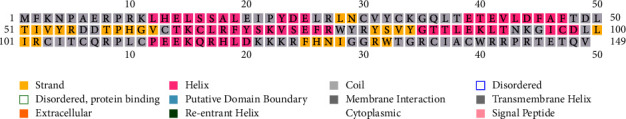
The secondary structure of HPV31 E6 protein.

**Figure 2:**
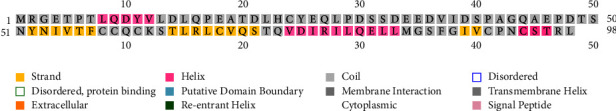
The secondary structure of HPV31 E7 protein.

**Figure 3:**
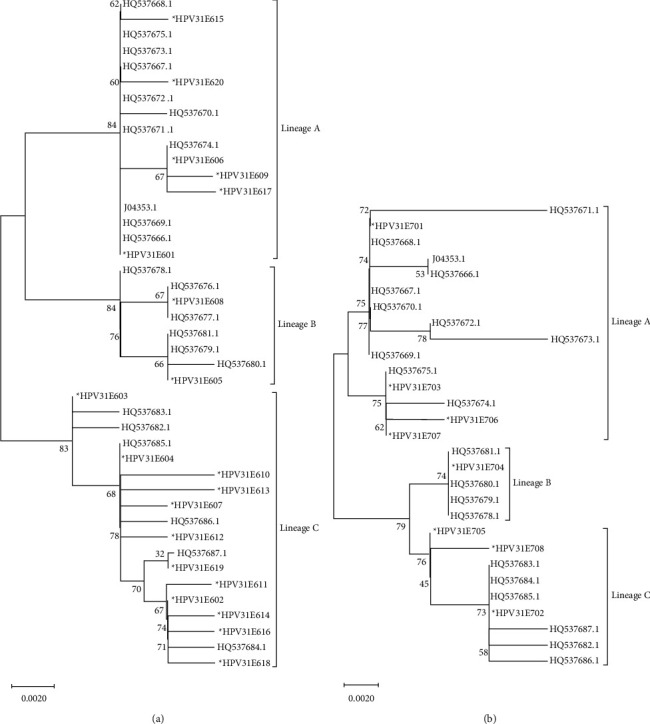
(a) The neighbor-joining tree of HPV31E6. ∗represents the sequences obtained in this study. Others were the variants reported previously in NCBI. Numbers above the branches are the bootstrap values. (b) The neighbor-joining tree of HPV31E7.

In the E7 gene, we found 8 (8/297) nucleotide positions that differed from the reference. 3 (37.50%) of them were synonymous substitutions while 5 (62.50%) were nonsynonymous. The isolates were divided into 8 subtypes named HPV31E701–HPV31E708, based on their mutations. No isolates showed complete homology with the reference. HPV31E707–HPV31E709 were found for the first time. All nucleotide variations and corresponding amino acid changes of the HPV31 E6 and E7 genes are listed in Tables 2 and 3. The results showed that E7 was clearly more conserved than E6. None of the mutations resulted in a frameshift or a premature stop codon. The secondary structures of the HPV31 E6 and E7 proteins are shown in Figures 1 and 2, while the secondary structures associated with the nonsynonymous mutations of HPV31 E6 and E7 are listed in Tables 2 and 3. Among the 151 HPV31 E6 sequences, lineage A (74,49.01%) had the highest frequency, followed by lineage C (62,41.06%) and lineage B (15,9.93%) (Table 2 and Figure 3(a)). In addition, among the 151 HPV31E7 sequences, lineage A (74,49.01%) showed the highest frequency, followed by lineage C (71,47.02%) and lineage B (6,3.97%) (Table 3 and Figure 3(b)).

### 3.2. Genetic Polymorphism of HPV33 E6 and E7

Upon comparison with the HPV33 reference sequence (M12732.1), 95 (69.85%) isolates were completely identical, while the remaining 41 (30.15%) showed single-nucleotide variations in the E6 gene. We found 17 (17/450) nucleotide positions that varied from the reference sequence. 7 (41.18%) of them were synonymous substitutions, while 10 (58.82%) were nonsynonymous. In the E6 gene, we found 1 triallelic mutation, which was a nonsynonymous mutation, leading to the amino acid change T5I/S. The isolates were divided into 14 subtypes, named HPV33E601–HPV33E614, based on their mutations. HPV31E601 showed complete homology with the reference, while HPV33E604, HPV33E605, HPV33E607, HPV33E608, HPV33E610, HPV33E612, and HPV33E614 were found for the first time.

**Figure 4:**
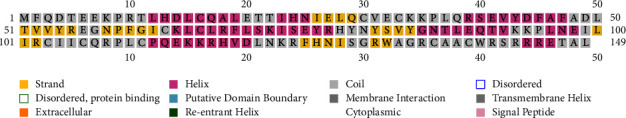
The secondary structure of HPV33 E6 protein.

**Figure 5:**
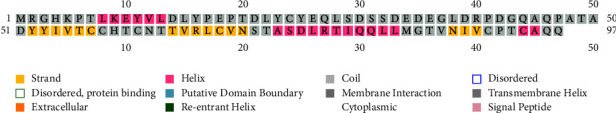
The secondary structure of HPV33 E7 protein.

In the E7 gene, we found 9 (9/294) nucleotide positions that differed from the reference. 3 (33.33%) of them were synonymous substitutions, while 6 (66.67%) were nonsynonymous. In this gene, we also found 2 triallelic mutations. Both of them were nonsynonymous mutations, leading to the amino acid changes A45V/E and T93S/N. The isolates were divided into 9 subtypes, named HPV33E701–HPV33E709, based on their mutations. HPV33E701 showed complete homology with the reference. The number of HPV33E701 cases was 103 (75.74%). HPV33E706, HPV33E708 and HPV33E709 were found for the first time. All nucleotide variations and corresponding amino acid changes of the E6 and E7 genes of HPV33 are listed in Tables 4 and 5. The E7 gene of HPV33 was more conserved than E6, as was the case for HPV31. None of the mutations produced a frameshift or a premature stop codon. The secondary structures of the E6 and E7 proteins of HPV33 are shown in Figures 4 and 5, while the secondary structures associated with the nonsynonymous mutations in E6 and E7 of HPV33 are listed in Tables 4 and 5.

**Figure 6:**
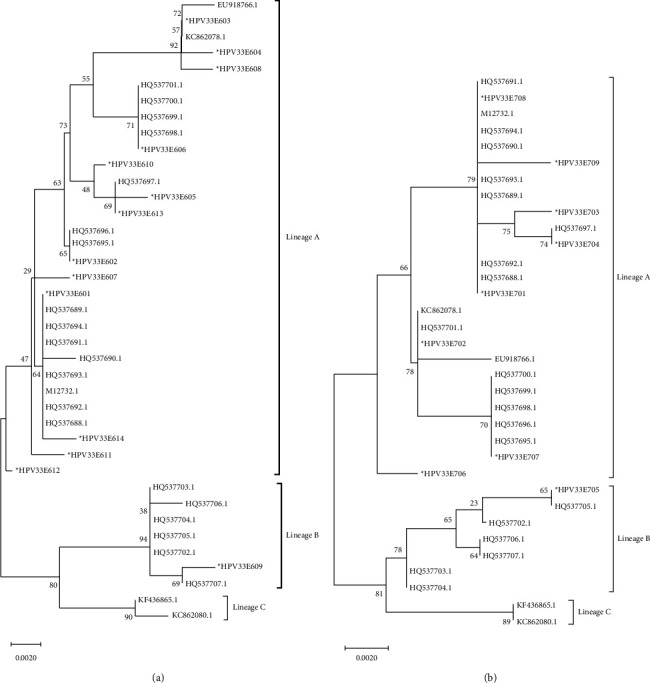
(a) The neighbor-joining tree of HPV33E6. (b) The neighbor-joining tree of HPV33E7.

Among the 136 HPV33 E6 sequences, lineage A (135,99.26%) was the most common, followed by lineage B (1,0.74%), while no sequences obtained in this study belonged to lineage C (Table 4 and Figure 6(a)). Among the 136 HPV33 E7 sequences, lineage A (135,99.26%) was the most common, followed by lineage B (1,0.74%), while again no sequences obtained here belonged to lineage C (Table 5 and Figure 6(b)).

### 3.3. Gene Sequences and Histological Characteristics

Among the 151 sequences of the E6 and E7 genes of HPV31 and the 136 such sequences of HPV33, 115 and 108 samples underwent histological diagnoses, respectively. The associations of the identified variants with cervical disease are shown in Tables 6–9. We only found a statistically significant association between the identified variants and cervical disease in HPV33E6. The different HPV33E6 sequences differ in terms of their potential for associations with cervical pathogenesis. However, more data on this issue are needed.

### 3.4. Selective Pressure Analysis

We used PAML4.9 software to test for variation in the dN/dS ratios among the different lineages detected in this study. K93N (*P* > 95%) in HPV33 E6 was the only positively selected site that we identified in this study. No positively selected sites were found in E6 and E7 of HPV31 or in E7 of HPV33.

### 3.5. Predicted MHC and B-Cell Epitopes

**Figure 7:**
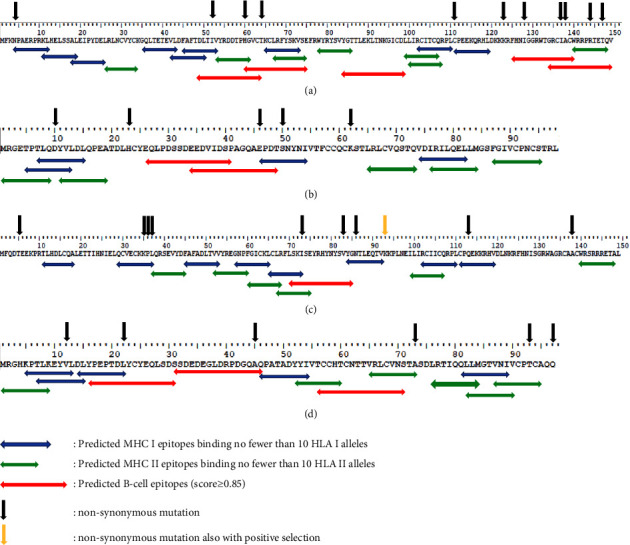
Predicted MHC and B-cell epitopes. (a) HPV31 E6 protein; (b) HPV31 E7 protein; (c) HPV33 E6 protein; (d) HPV33 E7 protein.

The MHC epitopes and B-cell epitopes were predicted and are shown in Figure 7; only MHC epitopes binding no fewer than 10 HLA class alleles are shown, and only B-cell epitopes scoring ≥0.85 are shown). For MHC I, we found 9 good epitopes in HPV31E6, 4 in HPV31E7, 8 in HPV33E6, and 5 in HPV33 E7. For MHC II, we found 7 good epitopes in HPV31E6, 5 in HPV31E7, 6 in HPV33E6, and 6 in HPV33 E7. For B-cell epitopes, 5 high-scoring epitopes were found in HPV31E6, 2 in HPV31E7, 1 in HPV33E6, and 3 in HPV33E7. All of the results are shown in Figure 7. The nonsynonymous mutations may cause changes in epitopes' binding abilities.

## 4. Discussion

Persistent infection with HR-HPV is a key factor associated with cervical cancer. HPV16 and HPV18 are the most common types of HPV worldwide. A large number of studies have shown that polymorphisms in the E6 and E7 genes of HPV16 and HPV18 are of great significance for the biological function of viruses and clinical disease progression. However, few studies on polymorphisms in the E6 and E7 genes of HPV31 and HPV33 have been carried out. This study provided an overview of these polymorphisms in northeast China.

This study showed that the mutation frequency of HPV31E6 is higher than that of HPV33E6, while the opposite pattern was identified in E7. In addition, the E7 gene is more conserved than E6 in both HPV31 and HPV33. Considering the previous findings that conservation of the E7 gene is critical to the association of HPV16 and HPV18 with carcinogenesis [19], E7 can be considered a more appropriate target for the diagnosis of HPV31 and HPV33 than E6. The nonsynonymous mutations leading to changes in the secondary structures of E6 and E7 proteins in HPV31 and HPV33 may result in changes to polarity, hydrophobicity, and amino acid side chains, which would potentially alter coprotein folding [20]. Therefore, the nonsynonymous mutations may affect the carcinogenic potential of the virus, the immune reaction of the host, and the potency of vaccines by altering the structure of E6 and E7 oncoproteins. In HPV31E6, C413T (A138V) was the most common nonsynonymous mutation. In HPV31E7, A184G (K62E) was the most common nonsynonymous mutation. In HPV33E6, 105C (K35N) was the most common nonsynonymous mutation. Finally, in HPV33E7, A190T (Q97L) was the most common nonsynonymous mutation. These mutations should be taken into account when E6 and E7 are chosen as targets for primer design or diagnosis. In this study, 10HPV31E6 sequences (HPV31E609−HPV31E614, HPV31E616−HPV31E618, and HPV31E620), 3HPV31E7 sequences (HPV31E707–HPV31E709), 7HPV33E6 sequences (HPV33E604, HPV33E605, HPV33E607, HPV33E608, HPV33E610, HPV33E612, and HPV33E614), and 3HPV33E7 sequences (HPV33E706, HPV33E708, and HPV33E709) were found for the first time. For further functional research and study of the potential for cervical pathogenesis, there is still a need for a large number of samples to be analyzed and many more experiments to be performed.

Xi et al. reported that HPV31 lineage A (41.7%) is the most common lineage in women in the USA, followed by lineage C (37.2%) and lineage B (21.1%) [21]. Our study showed the same distribution in northeast China. However, in Italy, lineage C (65.8%) was found to be the most common, followed by lineage B (29.3%), and lineage A (4.9%) [22]. Therefore, there is a need to bear in mind the possibility of geographic variation and ethnic differences in the frequencies of these lineages. Chen et al. also reported that HPV33 lineage A was the main lineage in Asia [23], which matches our study. In addition, here we only found one positively selected site, K93N in HPV33E6. This site was also previously identified by Chen et al. [13] in southeast China. K93N has also been observed in HPV58E6, so it may have evolutionary significance in enabling viruses to adapt to the environment and improve their survival.

Amino acid positions 145–149 form the PDZ binding domain in the E6 protein; amino acid positions 21–29 form a short linear motif responsible for Rb binding in the E7 protein; whereas positions 58, 61, 91, and 94 act as Zn binding sites in the E7 protein [23, 24]. In this study, HPV31E6 C430T (R144C) was observed next to residues 145–149, and A439G (T147A) was observed in residues 145–149. HPV31E7 C67T (H23Y) was observed in residues 21–29, and A184G (K62E) was observed beside residue 61. Moreover, HPV33E7 T65C (L22P) was found in residues 21–29, and C278G/A (T93S/N) was found beside residue 94. These mutations may alter the structures of oncoproteins, which could in turn affect the carcinogenic potential of viruses, the immune reaction of the host, and the potency of vaccines.

Modern immunoinformatics provides new strategies for the design and identification of antigen-specific epitopic sites for use as vaccine targets. Nearly all of the nonsynonymous mutations observed in this study are located in MHC I/II and/or B-cell epitopes. These nonsynonymous mutations may decrease or increase epitope binding ability, or even make epitopes appear or disappear. They can also make the scores of predicted B-cell epitopes lower or higher. Therefore, these nonsynonymous mutations may influence the interplay between viruses and host cells. The epitopes predicted in this study may contribute to the development of vaccines against specific HPV variants in the Chinese. However, further experiments are still required to confirm the predictions obtained through immunoinformatics.

This is the first study on the genetic polymorphism of the HPV31 and HPV33 E6 and E7 genes in northeast China. The presented data provide deeper insights into the diverse geographical distribution of HPV31 and HPV33 E6 and E7 genes and should contribute to the development and application of detection probes, targeted drugs, and vaccines against specific HPV variants in the Chinese.

## Figures and Tables

**Table 1:** Primers used for the molecular characterization of HPV31 and HPV33 E6 and E7.

Primer name	Sequence primers
HPV31 E6/E7 F1	5′-TAAACTGCCAAGGTTGTG-3′
HPV31 E6/E7 R1	5′-TGTTCCTCCGCTTCCTGT-3′
HPV31 E6/E7 F2	5′-AAAAGTAGGGAGTGACCG-3′
HPV31 E6/E7 R2	5′-TTCGTCCTCTGAAATGTTG-3′
HPV33 E6/E7 F1	5′-AAAAGTAGGGTGTAACCG-3′
HPV33 E6/E7 R1	5′-CTCTAGTAAATCCGTGCC-3′
HPV33 E6/E7 F2	5′-AGGTACTGCACGACTATGT-3′
HPV33 E6/E7 R2	5′-GTGCCACTGTCATCTGCT-3′

F, forward; R, reverse.

**Table 2:** Nucleotide sequence mutations of HPV31E6.

Category	Nucleotide position																									*n* (151)	Lineage
	11	21	27	69	81	90	141	154	178	190	202	207	213	219	297	321	331	368	375	383	391	409	413	430	439		
Reference J04353.1	A	A	T	C	A	T	T	A	C	A	A	T	A	A	G	A	T	A	C	T	A	A	C	C	A		A
HPV31E601	—	—	—	—	—	—	—	—	—	—	—	—	—	—	—	—	—	—	—	—	—	—	—	—	—	16	A
HPV31E602	—	—	—	—	—	—	—	—	T	—	—	—	T	G	A	G	—	—	—	—	—	—	T	—	—	21	C
HPV31E603	—	—	—	—	—	—	—	—	T	—	—	—	T	—	A	—	—	—	—	—	—	—	T	—	—	2	C
HPV31E604	—	—	—	—	—	—	—	—	T	—	—	—	T	—	A	G	—	—	—	—	—	—	T	—	—	29	C
HPV31E605	—	—	—	—	—	—	C	—	—	G	—	—	T	—	—	—	—	G	—	—	—	—	T	—	—	6	B
HPV31E606	—	—	—	T	—	—	—	—	—	—	—	—	—	—	—	—	—	—	—	—	—	—	—	—	—	49	A
HPV31E607	—	—	—	—	—	—	—	—	T	—	—	—	T	—	A	G	—	—	—	—	—	G	T	—	—	1	C
HPV31E608	—	—	—	—	—	—	—	C	—	G	—	—	T	—	—	—	—	G	—	—	—	—	T	—	—	9	B
HPV31E609	—	—	—	T	—	—	—	—	—	—	—	—	—	—	—	—	—	—	T	—	—	—	—	—	—	1	A
HPV31E610	—	—	—	—	G	—	—	—	T	—	—	—	T	—	A	G	—	—	—	—	—	—	T	—	G	1	C
HPV31E611	—	—	—	—	—	—	—	—	T	—	—	—	T	G	A	G	—	—	T	—	—	—	T	—	—	1	C
HPV31E612	—	—	—	—	—	—	—	—	T	—	—	—	T	—	A	G	C	—	—	—	—	—	T	—	—	1	C
HPV31E613	—	G	—	—	—	—	—	—	T	—	—	—	T	—	A	G	—	—	—	C	—	—	T	—	—	1	C
HPV31E614	—	—	—	—	—	C	—	—	T	—	—	—	T	G	A	G	—	—	—	—	—	—	T	—	—	1	C
HPV31E615	—	—	A	—	—	—	—	—	—	—	—	—	—	—	—	—	—	—	—	—	—	—	—	—	—	6	A
HPV31E616	—	—	—	—	—	—	—	—	T	—	—	C	T	G	A	G	—	—	—	—	—	—	T	—	—	1	C
HPV31E617	—	—	—	T	—	—	—	—	—	—	—	—	—	—	—	—	—	—	—	—	—	—	—	T	—	1	A
HPV31E618	—	—	—	—	—	—	—	—	T	—	—	—	T	G	A	G	—	—	—	—	C	—	T	—	—	1	C
HPV31E619	—	—	—	—	—	—	—	—	T	—	C	—	T	G	A	G	—	—	—	—	—	—	T	—	—	2	C
HPV31E620	C	—	—	—	—	—	—	—	—	—	—	—	—	—	—	—	—	—	—	—	—	—	—	—	—	1	A
aa mutation	Amino acid position																										
	4	7	9	23	27	30	47	52	60	64	68	69	71	73	99	107	111	123	125	128	131	137	138	144	147		
Reference J04353.1	N	E	P	Y	R	C	F	I	H	T	R	F	S	V	L	Q	C	K	F	I	R	I	A	R	T		
	T	—	—	—	—	—	—	L	Y	A	—	—	—	—	—	—	R	R	—	T	—	V	V	C	A		
Protein secondary structure	C							E	E	H							C	C		E		C	C	C	C		

Dashes (−) represent that nucleotides are identical to the reference sequence. Nucleotide mutations are presented by the corresponding letters. aa, amino acid. “*n*” represents the number of each species among the samples examined. C, random coil; E, extended strand; H, alpha helix.

**Table 3:** Nucleotide sequence mutations of HPV31E7.

Category	Nucleotide position								*n* (151)	Lineage
	21	29	67	111	136	149	184	228		
Reference J04353.1	G	A	C	C	G	C	A	T		A
HPV31E701	—	—	—	—	—	—	G	—	3	A
HPV31E702	A	—	—	T	A	—	G	—	62	C
HPV31E703	—	—	T	—	—	—	G	—	69	A
HPV31E704	—	—	T	T	A	—	G	—	6	B
HPV31E705	—	—	—	T	A	—	G	—	8	C
HPV31E706	—	G	T	—	—	—	G	—	1	A
HPV31E707	—	—	T	—	—	—	G	C	1	A
HPV31E708	—	—	—	T	A	A	G	—	1	C
aa mutation	Amino acid position									
	7	10	23	37	46	50	62	76		
Reference J04353.1	T	D	H	V	E	S	K	I		
	—	G	Y	—	K	Y	E	—		
Protein secondary structure		H	C		C	C	C			

**Table 4:** Nucleotide sequence mutations of HPV33E6.

Category	Nucleotide position																	*n* (136)	Lineage
	14^*∗*^	18	96	105	106	109	165	207	217	247	256	279	338	372	413	426	441		
Reference M12732.1	C	G	A	A	C	T	A	C	A	G	A	A	A	A	C	C	T		A
HPV33E601	—	—	—	—	—	—	—	—	—	—	—	—	—	—	—	—	—	95	A
HPV33E602	—	—	—	C	—	—	—	—	—	—	—	—	—	—	—	—	—	12	A
HPV33E603	—	—	—	C	—	—	G	—	—	—	C	C	G	—	—	—	—	12	A
HPV33E604	T	—	—	C	—	—	G	—	—	—	C	C	G	—	—	—	—	1	A
HPV33E605	G	—	—	C	—	—	—	—	—	—	—	C	—	—	—	—	C	1	A
HPV33E606	—	—	—	C	—	—	—	—	—	—	C	—	—	T	—	—	—	5	A
HPV33E607	—	—	—	—	—	G	—	—	—	—	—	—	—	—	—	—	—	2	A
HPV33E608	—	—	—	C	—	G	G	—	—	—	C	C	G	—	—	—	—	1	A
HPV33E609	—	—	G	—	A	—	—	T	C	T	—	C	—	—	T	T	—	1	B
HPV33E610	—	—	—	C	—	—	—	—	—	—	—	—	—	—	—	—	C	2	A
HPV33E611	—	—	—	—	A	—	—	—	—	—	—	—	—	—	—	—	—	1	A
HPV33E612	—	—	—	—	—	—	—	—	—	—	—	—	—	—	T	—	—	1	A
HPV33E613	—	—	—	C	—	—	—	—	—	—	—	C	—	—	—	—	C	1	A
HPV33E614	—	A	—	—	—	—	—	—	—	—	—	—	—	—	—	—	—	1	A
aa mutation	Amino acid position																		
	5	6	32	35	36	37	55	69	73	83	86	93	113	124	138	142	147		
Reference M12732.1	T	E	E	K	P	L	R	F	I	V	N	K	Q	R	A	S	T		
	I/S	—	—	N	T	V	—	—	L	L	H	N	R	—	V	—	—		
Protein secondary structure	C			C	C	C			H	E	H	C	H	C	C				

^*∗*^indicates the positions of triallelic mutations.

**Table 5:** Nucleotide sequence mutations of HPV33E7.

Category	Nucleotide position									*n* (136)	Lineage
	34	65	134^*∗*^	150	165	218	261	278^*∗*^	290		
Reference M12732.1	G	T	C	T	A	C	G	C	A		A
HPV33E701	—	—	—	—	—	—	—	—	—	103	A
HPV33E702	—	—	—	—	—	—	—	—	T	13	A
HPV33E703	—	—	T	—	—	—	—	—	—	7	A
HPV33E704	—	—	A	—	—	—	—	—	—	4	A
HPV33E705	A	—	—	C	—	—	T	G	T	1	B
HPV33E706	—	—	—	—	—	—	—	A	T	2	A
HPV33E707	—	—	—	—	G	—	—	—	T	4	A
HPV33E708	—	—	—	—	—	T	—	—	—	1	A
HPV33E709	—	C	—	—	—	—	—	—	—	1	A
aa mutation	—	—	—	—	—	—	—	—	—		
	12	22	45	50	55	73	87	93	97		
Reference M12732.1	V	L	A	A	V	A	V	T	Q		
	I	P	V/E	—	—	V	—	S/N	L		
Protein secondary structure	H	C	C			H		C	C		

**Table 6:** The associations of HPV31E6 variants with cervical disease.

	Grade of related cervical lesion					
	Sample = 115	Normal = 61	Cervicitis = 10	CIN 1 = 17	CIN 2Y3 = 26	CC = 1
HPV31E601	13	5	0	3	5	0
HPV31E602	17	10	3	2	2	0
HPV31E603	1	1	0	0	0	0
HPV31E604	23	12	2	4	5	0
HPV31E605	4	1	2	1	0	0
HPV31E606	33	16	3	4	10	0
HPV31E607	0	0	0	0	0	0
HPV31E608	8	4	0	3	0	1
HPV31E609	0	0	0	0	0	0
HPV31E610	1	1	0	0	0	0
HPV31E611	1	1	0	0	0	0
HPV31E612	1	1	0	0	0	0
HPV31E613	1	1	0	0	0	0
HPV31E614	1	1	0	0	0	0
HPV31E615	5	1	0	0	4	0
HPV31E616	1	1	0	0	0	0
HPV31E617	1	1	0	0	0	0
HPV31E618	1	1	0	0	0	0
HPV31E619	2	2	0	0	0	0
HPV31E620	1	1	0	0	0	0

CIN2Y3 indicates CIN2 or CIN3; CC indicates cervical cancer.

**Table 7:** The associations of HPV31E7 variants with cervical disease.

	Grade of related cervical lesion					
	Sample = 115	Normal = 63	Cervicitis = 8	CIN 1 = 18	CIN 2Y3 = 26	CC = 1
HPV31E701	0	0	0	0	0	0
HPV31E702	2	0	0	1	2	0
HPV31E703	50	33	4	7	6	0
HPV31E704	50	24	2	6	18	0
HPV31E705	4	1	2	1	0	0
HPV31E706	7	4	0	3	0	0
HPV31E707	0	0	0	0	0	0
HPV31E708	1	1	0	0	0	0
HPV31E709	1	0	0	0	0	1

**Table 8:** The associations of HPV33E6 variants with cervical disease.

	Grade of related cervical lesion					
	Sample = 108	Normal = 49	Cervicitis = 10	CIN 1 = 11	CIN 2Y3 = 38	CC = 0
HPV33E601	74	30	7	6	31	0
HPV33E602	10	3	1	3	3	0
HPV33E603	10	6	2	0	2	0
HPV33E604	1	1	0	0	0	0
HPV33E605	1	1	0	0	0	0
HPV33E606	5	5	0	0	0	0
HPV33E607	2	1	0	1	0	0
HPV33E608	0	0	0	0	0	0
HPV33E609	1	0	0	0	1	0
HPV33E610	1	0	0	1	0	0
HPV33E611	0	0	0	0	0	0
HPV33E612	1	1	0	0	0	0
HPV33E613	1	0	0	0	1	0
HPV33E614	1	1	0	0	0	0

**Table 9:** The associations of HPV33E7 variants with cervical disease.

	Grade of related cervical lesion					
	Sample = 108	Normal = 49	Cervicitis = 10	CIN 1 = 11	CIN 2Y3 = 38	CC = 0
HPV33E701	80	31	8	10	31	0
HPV33E702	10	7	1	0	2	0
HPV33E703	6	3	0	0	3	0
HPV33E704	4	2	0	1	1	0
HPV33E705	1	0	0	0	1	0
HPV33E706	2	1	1	0	0	0
HPV33E707	4	4	0	0	0	0
HPV33E708	0	0	0	0	0	0
HPV33E709	1	1	0	0	0	0

## Data Availability

The data used to support the findings of this study are included within the article.
